# Pan-Cancer Analysis of Mutations Affecting Protein Liquid–Liquid Phase Separation Revealing Clinical Implications

**DOI:** 10.3390/biology14101320

**Published:** 2025-09-25

**Authors:** Xiaoping Cen, Lulu Wang, Kai Yu, Huanming Yang, Roland Eils, Wei Dong, Huan Lin, Zexian Liu

**Affiliations:** 1 State Key Laboratory of Oncology in South China, Collaborative Innovation Center for Cancer Medicine, Sun Yat-sen University Cancer Center, Guangzhou 510060, China; cenxiaoping20@mails.ucas.ac.cn (X.C.); wangll2@sysucc.org.cn (L.W.); kaiyu9510@gmail.com (K.Y.); 2 Digital Health Center, Berlin Institute of Health (BIH), Charité—Universitätsmedizin Berlin, 10178 Berlin, Germany; roland.eils@bih-charite.de; 3 College of Life Sciences, University of Chinese Academy of Sciences, Beijing 100049, China; yanghm@genomics.cn; 4 HIM-BGI Omics Center, Hangzhou Institute of Medicine (HIM), Chinese Academy of Sciences (CAS), Zhejiang Cancer Hospital, Hangzhou 310022, China; dongw@bgi.com; 5 BGI, Shenzhen 518083, China; 6 James D. Watson Institute of Genome Sciences, Hangzhou 310029, China; 7 Intelligent Medicine Institute, Fudan University, Shanghai 200032, China; 8 Clin Lab, BGI Genomics, Beijing 100000, China; 9 The Affiliated TCM Hospital of Guangzhou Medical University, Guangzhou 510020, China

**Keywords:** phase separation, pan-cancer, mutations, landscape

## Abstract

Phase separation is one of the mechanisms critical for protein function, and its aberrances are associated with cancer development. However, mutations that affect protein phase separation and cancer development have not been systematically identified and analyzed. In this study, we systematically identified the mutations affecting protein liquid–liquid phase separation in multiple cancers. We calculated the phase separation scores alterations for over 1,200,000 mutations across 16 cancer types using the TCGA dataset. We then performed pathway enrichment, kinase, TF enrichment, and survival analysis to identify related biological processes and clinical implications. Nearly 10% of the mutations were defined to affect phase separation in pan-cancer. These mutations occupied a consistent percentage in each cancer type. Extremely influencing mutations accumulate on stomach adenocarcinoma (STAD), uterine corpus endometrial carcinoma (UCEC), and skin cutaneous melanoma (SKCM). Moreover, proteins carrying these mutations are enriched in cancer-related pathways, including TGF-beta signaling pathways and polycomb repressive complex. Phase separation of these proteins would be regulated by kinases, including CDK1, CDK2, and EGFR, and transcription factors, including ZNF407, ZNF318, and MGA proteins, to play functions in cancer. Protein–Protein Interaction Network revealed that these phase separation proteins are highly interconnected. Finally, patients carrying mutations that positively affect the protein phase separation are associated with poor prognosis in skin cutaneous melanoma (SKCM) and lung squamous cell carcinoma (LUSC), which could be partially explained by the pathogenicity of these mutations. The study provided a pan-cancer landscape for depicting the association of phase separation and cancer mutations, which would be a rich data resource for understanding the association of cancer mutations and phase separation.

## Simple Summary

Identifying mutations affecting protein phase separation is important for understanding cancer development. This research provided a pan-cancer landscape of mutations affecting protein liquid–liquid phase separation. The researchers calculated the phase separation scores alterations for 1,267,908 mutations across 16 cancer types using the TCGA dataset. Nearly 10% of the mutations were phase separation-affecting mutations in pan-cancer dataset. Proteins carrying these mutations were enriched in cancer-related pathways, including TGF-beta signaling pathways and polycomb repressive complex. Phase separation of these proteins would be regulated by kinases, including CDK1, CDK2, and EGFR, and transcription factors, including ZNF407, ZNF318, and MGA proteins, to play functions in cancer. Protein–Protein Interaction Network revealed that these phase separation proteins are highly interconnected. Finally, patients carrying mutations that positively affect the protein phase separation are associated with poor prognosis in skin cutaneous melanoma (SKCM) and lung squamous cell carcinoma (LUSC). This research provided a pan-cancer landscape for depicting the association of phase separation and cancer mutations, revealing clinical implications for phase separation.

## 1. Introduction

Phase separation (PS), also named liquid–liquid phase separation (LLPS), is a phenomenon in which biomolecules form phase-separated condensates. The LLPS of protein is suggested to be associated with the multivalency of the protein, which is often characterized by the presence of intrinsically disordered regions (IDRs) [1,2]. Phase-separating proteins play important functions in normal physiological conditions, including transcription regulation and genome organization construction. For example, NELF (Negative Elongation Factor) protein can form condensates to suppress the transcription of housekeeping genes and maintain cell survival [3]. Protein phase separation induces the generation of subcellular structures in cells, such as membraneless organelles [1,4,5] and heterochromatin [6,7]. Many biological pathways, including signal transduction, RNA metabolism, and autophagy, are related to phase separation [8,9,10,11]. Studies have suggested that phase separation is related to tumorigenesis. One mechanism is that phase separation drives aberrant chromatin looping to induce cancer development [12]. Another kind of association is focused on IDR mutations and aberrant phase separation. Genetic variations, especially mutations on IDRs, are associated with dysregulated phase separation in key cellular processes and potentially cause cancer development [13]. However, to what extent cancer mutations affect protein phase separation, as well as the related biological pathways involved in this process, remains largely unknown.

Identifying the PS regions of a given protein sequence is important to define whether a protein could undergo phase separation. However, annotated information for phase-separating proteins was lacking, and to what extent the mutations could affect protein phase separation could not be obtained. The protein sequence information is important to determine the LLPS properties of proteins, whereas various software programs have been developed to identify the phase separation regions and calculate the phase separation scores for a given protein sequence [14,15,16,17]. Among them, dSCOPE [17], our previously developed software, has also achieved satisfactory performance. However, no study to date has identified the role of phase separation-affecting mutations in cancer development.

Several studies have indicated the role of phase separation in different cancer types. For example, Liu et al. identified phase separation-related gene signatures in predicting the prognosis of skin cutaneous melanoma [18]. And Sun et al. stratified molecular subtypes for bladder cancer with distinct prognoses [19]. Additionally, Zou et al. performed pan-cancer analysis on the mutational landscape of intrinsically disordered protein regions (IDRs) and revealed potential driver genes enriched in IDRs [20]. The study suggested that IDRs exhibit higher mutation frequencies across cancers and that genes enriched with IDR mutations were associated with phase separation, which indicated potential correlations of mutations with protein phase separation. While most studies focused on mutations in IDR regions, PSMutPred indicated the role of mutations in folded regions in affecting phase separation [21]. However, existing studies on mutations affecting phase separation focused on rare genetic diseases and Alzheimer’s disease, while neglecting the investigation in oncology.

In order to comprehensively depict the associations of mutations affecting phase separation with cancer development, we applied dSCOPE to define mutations affecting phase separation among 1,200,000 cancer mutations on a pan-cancer dataset, identifying potential molecular mechanisms and clinical relevance.

## 2. Materials and Methods

**Figure 1:**
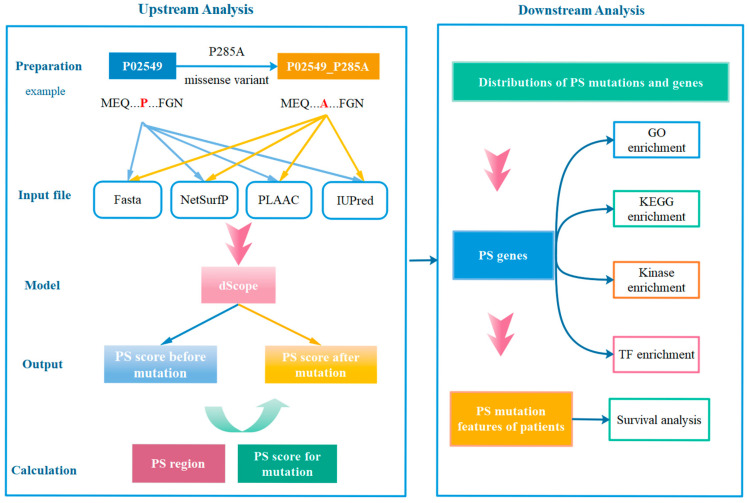
The workflow of the study.

The workflow of the present study is summarized in Figure 1.

### 2.1. Somatic Mutations Among Various Cancers

Somatic mutations across cancers were obtained from The Cancer Genome Atlas (TCGA) (https://xenabrowser.net/datapages/ accessed on 1 December 2024). Only missense mutations were included in this study. To save computation time and ensure visualization effect, the top 16 cancer types with high mutation number were involved, including UCEC, SKCM, COAD, LUAD, STAD, LUSC, BLCA, BRCA, HNSC, GBM, CESC, OV, READ, LIHC, ESCA, and LGG (Supplementary Table S1). A total of 1,267,908 mutations were finally involved after duplication filtering. Missense mutations with benign effect from the humsavar index were downloaded (https://ftp.uniprot.org/pub/databases/uniprot/current_release/knowledgebase/variants/humsavar.txt accessed on 1 September 2025) and used for comparison.

### 2.2. Protein Sequences

The sequences of all 20,422 human-reviewed proteins with corresponding sequences were downloaded from UniProt (https://www.uniprot.org/ accessed on 1 December 2024). After alignment with the proteins with at least one mutation in the study, 17,874 sequences were applied for analysis. In this study, we calculated the phase separation score alteration for each single mutation in a protein sequence.

### 2.3. Identification of Phase-Separating Proteins

Two peptides were generated for each mutation, corresponding to the conditions before and after the mutation. Then, dSCOPE [17] (https://dscope.omicsbio.info/) software was applied to identify the PS region and calculate the PS score of each peptide. Specifically, as described in [17], we used the same software, NetSurfP-2.0 [22], PLAAC [23] (http://plaac.wi.mit.edu/), and IUPred [24] (https://iupred3.elte.hu/), to calculate the protein features for each amino acid as model input. The model built from dSCOPE was trained on 1737 PS-positive peptides and 3125 PS-negative peptides using the random forest algorithm, which was then used as a trained model in this study. The protein features of each peptide were fitted to the model to obtain the PS scores. During the modeling process, each peptide was split into small peptides with a sequence window of 15 amino acids. A cutoff of 0.5208 was used to define whether the peptides could be considered potential phase separation regions. The average PS scores of peptides were calculated using the mean values of all the peptides containing the corresponding mutations, which consider fairly the influence of mutations on the phase separation of neighborhood sequences. Then, the delta PS score was calculated for each mutation based on the difference between average PS score of wild-type peptides and mutant peptides.

### 2.4. Pathway Enrichment Analysis of Cancer-Related Genes

To identify the function of proteins with mutations that affect phase separation, GO and KEGG enrichment analyses were performed using the R package ClusterProfiler (https://bioconductor.org/packages/release/bioc/html/clusterProfiler.html accessed on 1 January 2025, version: 4.14.4) on the genes that harbor phase separation-affecting mutations for all cancer types and each cancer type.

### 2.5. Kinase and TF Enrichment Analysis

The corresponding kinase-substrate information was collected from Group-based Prediction System (GPS) 6.0 (http://gps.biocuckoo.cn/ accessed on 5 January 2025). Fisher’s exact test was performed to identify the enriched kinases of the phase-separating proteins for each cancer type. ChEA3 was utilized to perform transcription factor analysis. Protein–Protein Interaction (PPI) Network was performed using the STRING database and Cytoscape software 3.10.0.

### 2.6. Survival Analysis

We performed survival analysis to evaluate the association between phase separation-affecting mutations and the clinical characteristics of patients. Detailed clinical information of the 16 cancers was downloaded from the Xena webserver. Multivariable Cox regression models were conducted, and Kaplan‒Meier curves were generated using the R package survival (https://cran.r-project.org/web/packages/survival/ accessed on 10 January 2025, version: 3.7.0). The pathogenic effects of the mutations were analyzed based on the AlphaMissense [24] program.

## 3. Results

### 3.1. Distribution of Phase Separation-Affecting Mutations Among Cancer Types

**Figure 2:**
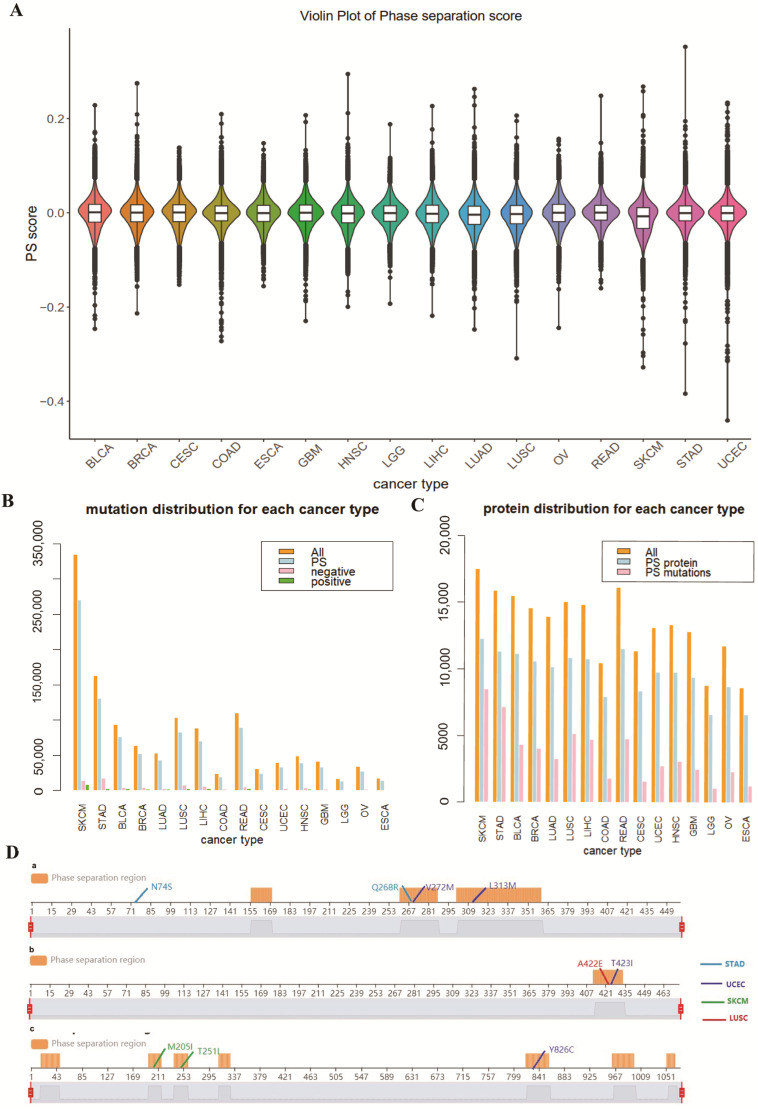
(**A**) Prevalent phase separation affected mutations across cancer types. (**B**) Distributions of different kinds of mutations or proteins in each cancer type. ‘PS’ refers to the mutations occurring on the predicted PS proteins, ‘negative’ and ‘positive’ refer to mutations that are considered to affect phase separation, with PS score difference <−0.05 or >0.05, respectively. (**C**) ‘PS mutations’ refers to the proteins that harbor phase separation-affecting mutations. (**D**) Top 10 mutations with the highest delta PS score on the three phase-separating proteins. Different colors represent the mutations that occurred in different corresponding cancer types. (**a**) ACD protein, (**b**) CALCR protein, (**c**) TMEM132E protein.

After calculating the phase score difference between mutated and wild-type proteins, we mapped the score alterations to the mutations that occurred in 16 cancer types. A statistical *t*-test indicated a significant difference between the PS score of wild-type proteins and of mutants (*p* < 0.001), suggesting that cancer mutations significantly affect the phase separation of the corresponding proteins. The delta PS score of benign mutations ranges from −0.1 to 0.1, while cancer mutations range from −0.44 to 0.35, suggesting statistical significance (*p* < 2.2 × 10^−16^, Supplementary Figure S1). Most cancer types carry mutations with delta PS score ranging from −0.3 to 0.2, while stomach adenocarcinoma (STAD), uterine corpus endometrial carcinoma (UCEC), and skin cutaneous melanoma (SKCM) have some extremely influencing mutations with delta PS score lower than −0.3 or higher than 0.2, indicating that the mutations in these cancer types are more likely to be relevant to the regulation of protein phase separation (Figure 2A).

As mutations associated with aberrant phase separation play important roles in disease development [25,26,27], we considered mutations that changed corresponding proteins’ PS score from <0.5208 to >0.5208 or vice versa, as mutations potentially cause aberrant phase separation. We found that the PS score alterations of these mutations are statistically different from the other mutations (*p* < 0.05), and the mean value of the PS score alterations of these mutations approaches 0.05. We then set a cutoff of 0.05 for all the mutations and regarding mutations with absolute PS score alterations over 0.05 as phase separation-affecting mutations, considering the broader meaning of phase separation-affecting mutations. Among all the mutations, 118,405 were considered to affect phase separation, which accounts for 10% of all mutations. Proteins with phase separation regions were considered phase-separating proteins, and we found 12,473 phase-separating proteins among 17,874 proteins, with an approximate percentage of 70%. We filtered the mutations occurring on non-PS proteins and calculated the type distribution of these mutations among different cancer types (Figure 2B). From Figure 2B,C, the mutations considered to affect phase separation occupied a considerable and consistent percentage in each cancer type, and the phase-separating protein occupied a high percentage in each cancer type. In addition, we found that mutations negatively affecting phase separation (delta PS score < −0.05) occupy a larger percentage than mutations that positively affect phase separation (delta PS score > 0.05). We also found that many of the mutations induced changes in the definition of protein phase separation regions. These results presented an important distribution of mutations affecting protein phase separation in cancer.

In order to further identify dominant mutations affecting protein phase separation, we first summarized the top ten mutations inducing the most considerable changes in phase separation scores. The mutations are mainly happening on predicted PS proteins, including ACD (Adrenocortical Dysplasia Protein), CALCR (Calcitonin Receptor), and TMEM132E (Transmembrane Protein 132E) proteins, and most of the mutations occurred in the PS regions of the corresponding proteins (Figure 2D). ACD protein is a shelterin protein involved in the maintenance of telomere length and in cancer radioresistance, whereas telomeres have been reported to be regulated by many mechanisms, including phase separation-dependent mechanisms [28]. CALCR protein and TMEM132E protein are transmembrane proteins, whereas phase separation can promote the assembly of transmembrane proteins with their cytoplasmic binding partners into micron-sized membrane-associated condensates [29].

We next analyzed whether the genes that harbor most mutations also harbor many PS mutations. The mutations are mostly distributed among different cancer types, indicating the heterogeneity of the mutation distribution among different cancer types. For example, the RYR2 protein is related to cardiac pacing, and mutation of the RYR2 gene is related to patient survival in non-small cell lung cancer [30]. Here, we found that the PS score of the RYR2 gene is dominantly enriched in two main subtypes of non-small cell lung cancer, lung adenocarcinoma (LUAD) and lung squamous cell carcinoma (LUSC)**.** The p53 protein is reported to undergo phase separation, which is consistent with our prediction result. Moreover, it acts as a tumor suppressor, and its mutants play an important role in promoting cancer development. Here, we also found that the p53 protein harbors a high percentage of mutations (11.35%) affecting protein phase separation.

### 3.2. GO and KEGG Enrichment Analysis for Genes Harboring Phase Separation-Affecting Mutations

**Figure 3:**
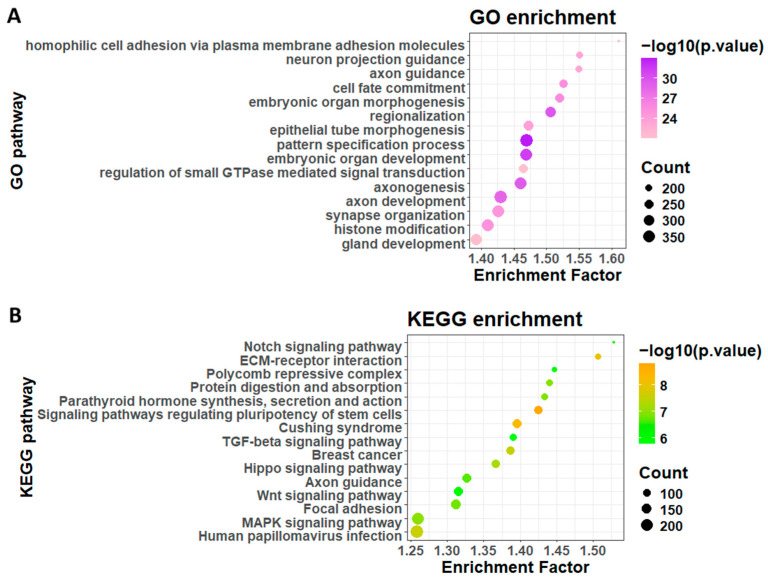
Pathway enrichment analysis of genes harboring phase separation-affecting mutations. (**A**) GO analysis of genes harboring phase separation-affecting mutations; (**B**) KEGG analysis of genes harboring phase separation-affecting mutations.

We next performed GO and KEGG analyses on specific gene sets of interest to identify pathways of phase separation in cancer development. Proteins with and without phase separation regions were compared by pathway enrichment analysis on the corresponding protein-coding genes. Phase-separating protein-coding genes were enriched in neurological processes and components, including axon development, synapse organization, and synaptic membrane, while non-phase-separation protein-coding genes were enriched in metabolic and ribosome-related processes and components (Supplementary Figure S2A,B). The KEGG pathways enriched in non-phase-separation protein-coding genes are mainly immunological pathways, including COVID-19 and drug metabolism pathways, while those in phase-separating protein-coding genes involve cancer pathways, including small cell lung cancer, breast cancer, and cancer-related signaling pathways, including Wnt, MAPK, and Hippo signaling pathways (Supplementary Figure S2C,D). Furthermore, we performed KEGG enrichment analysis on the defined genes (genes that harbor PS-affecting mutations), which involved more cancer-related pathways, including TGF-beta signaling pathways and polycomb repressive complex (Figure 3B). Polycomb repressive complex includes PRC1 and PRC2 proteins, which play important roles in cancer development. PCR1 protein was reported, and also predicted PS protein, which harbors PS-affecting mutations. These results validate the defined PS-affecting mutations and indicate important cancer pathways involved in the aberrant phase separation. We also calculated the mutations with PS score > 0.05 in the humsavar dataset, identifying 2273 mutations and 1653 genes. GO analysis suggested that these genes were enriched in DNA damage, DNA recombination pathways, and KEGG analysis identified that the genes were enriched in protein digestion and absorption, ECM–receptor interaction, and homologous recombination pathways (Supplementary Figure S3). These pathways are distinct from the pathways identified in the cancer dataset, indicating a different phase separation mechanism between cancer and benign samples.

Most reports on mutations affecting phase separation focus on IDR mutations, and here we sought to investigate the difference between the mechanisms of mutations in PS regions and outside of PS regions. A total of 115,850 mutations are on the PS regions of the corresponding protein, occupying 10% among all the mutations with PS score alterations. Genes carrying most mutations outside PS regions are mostly enriched in glycometabolic process (Supplementary Figure S4A), indicating that these mutations are related to the promotion of the LLPS of glycoproteins. Previous studies also indicated that the LLPS of glycogen is related to the tumorigenesis of liver cancer [31]. Mutations on PS regions are mostly enriched in epidermal cell differentiation (Supplementary Figure S4B), indicating the potential association of these mutations with the disruption of phase separation-based skin barrier governance and thereby associated with tumorigenesis [32], especially the tumorigenesis of skin cancer.

### 3.3. Protein-Level Analyses on Affected Phase-Separating Proteins

There is wide evidence on the interaction of kinases and phase separation and their role in cancer development. Kinases can act as scaffolds, co-drivers, and clients of LLPS, while LLPS could serve as an activation mechanism for oncogenic kinase fusions [2]. In addition, phase-separating proteins have been confirmed to regulate gene expression and activate transcription processes [33,34,35,36], and identifying transcription factors that lead to changes in gene expression is important to elucidate this process. Therefore, we performed kinase and TF enrichment analysis on the phase-separating proteins defined in the previous sections.

**Figure 4:**
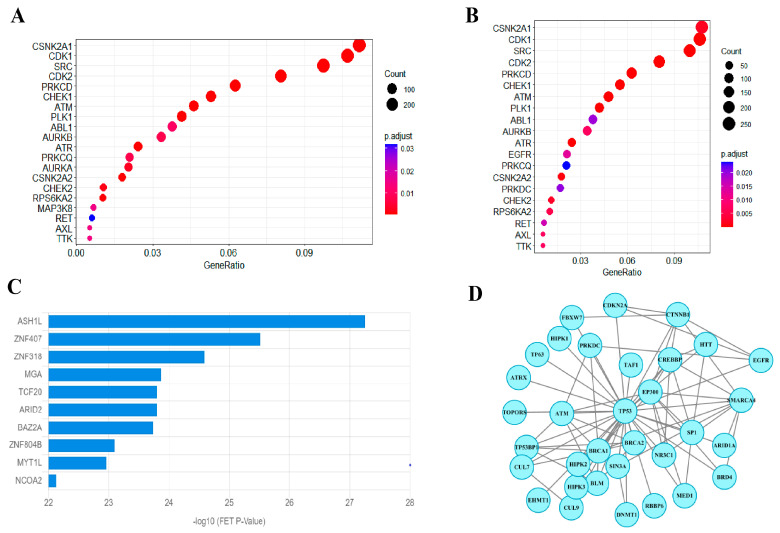
Analyses on phase-separating proteins. (**A**) Kinase enrichment analysis of all the predicted phase-separating proteins. (**B**) Kinase enrichment analysis of the defined phase-separating proteins. (**C**) TF enrichment analysis of the defined phase-separating proteins. (**D**) PPI subnetwork of the defined phase-separating proteins.

As shown in Figure 4A,B, kinase enrichment analysis indicated that phase-separating proteins were significantly enriched in CSNK2A1, SRC, CDK1, and CDK2. SRC has played a key role in the PS of the FUS and tau proteins [37,38], and several Src inhibitors have been approved by the FDA (Food and Drug Administration) for cancer treatments. CDK1 and CDK2 play roles in the cell cycle, and related inhibitors have also been extensively developed. Moreover, EGFR (epidermal growth factor receptor) is an important kinase that is involved in cancer cell growth. These results indicate the essential role of kinases in regulating LLPS and tumorigenesis.

Transcription factor (TF) enrichment analysis was performed using ChIP-X Enrichment Analysis 3 (ChEA3) [39]. As shown in Figure 4C, the top ten transcription factors (TFs) were ASH1L, ZNF407, ZNF318, MGA, TCF20, ARID2, BAZ2A, ZNF804B, MYT1L, and NCOA2. Among these enriched TFs, zinc-finger proteins occurred frequently. Zinc-finger proteins form the largest family of sequence-specific DNA-binding proteins, and many of the proteins are proven to be relevant to cancer progression. In addition, MGA protein is considered an important protein for cancer development; however, studies on the function of this protein are lacking. A recent study indicated that MGA protein could control the expression of BMPs (Bone Morphogenetic Proteins) gene [40], whereas BMPs play dual roles in suppressing tumor growth and metastasis and accelerating tumorigenesis [41].

Phase-separated proteins may harbor distinct biological characteristics in protein–protein interactions (PPIs) [42]. Therefore, we further performed PPI analysis based on the defined PS proteins. We found a number of other defined proteins that interact with the reported PS protein, p53 proteins (Figure 4D). This further indicates the role of mutations in affecting protein–protein interactions.

### 3.4. Survival Analysis of Phase Separation-Affecting Mutations in Cancer

To identify the associations of phase separation properties with patient prognosis, we calculated three variables for different patients in different cancer types: (1) mut_num, which refers to the number of mutations that meet the cutoff of 0.05 for each patient. (2) score_all, which refers to the sum of the changes in phase separation-affecting mutations; and (3) percent_mut, which refers to the percentage of mutations with PS score > 0 among all the mutations. To fairly reveal the role of these variables in patient prognosis, we performed a multivariate Cox regression analysis, which considered prognosis-related clinical factors (age, sex, tumor mutation burden (TMB), and tumor stage) as background. Then, we selected variables with *p* < 0.05 in a multivariate Cox regression model for each cancer. The median values were used as cutoffs to divide each variable into two groups, and Kaplan–Meier curves were plotted.

**Figure 5:**
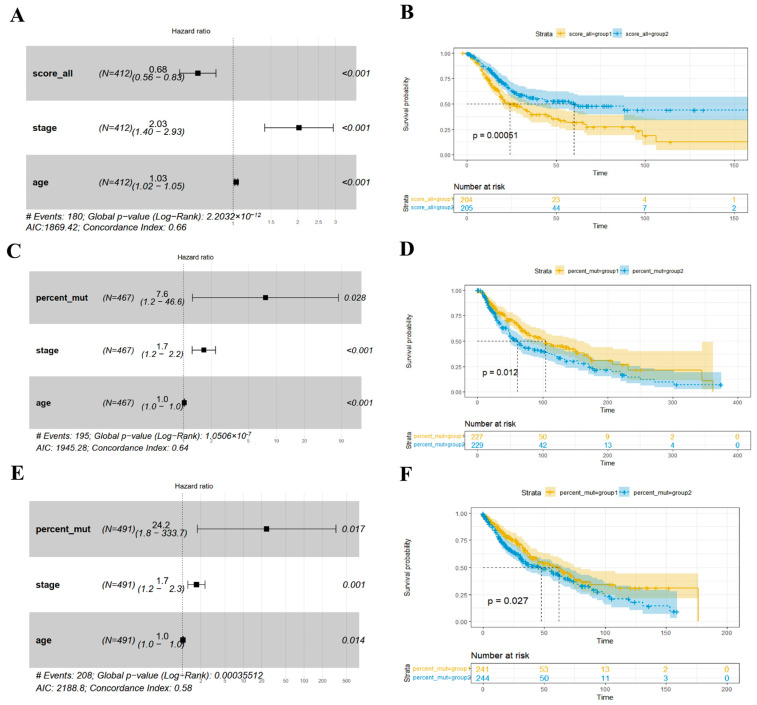
Survival analysis of phase separation-affecting mutations. (**A**,**B**) Multivariable Cox model and single-variable Kaplan‒Meier curve for BLCA; (**C**,**D**) multivariable Cox model and single-variable Kaplan‒Meier curve for SKCM; (**E**,**F**) multivariable Cox model and single-variable Kaplan‒Meier curve for LUSC.

From Figure 5, the percent_mut variable was selected from a multivariable model for SKCM and lung squamous cell carcinoma (LUSC). Kaplan‒Meier curves showed that a high percentage of mutations positively influencing phase separation was associated with poor prognosis. In addition, the score_all variable was also selected from a multivariable model for bladder urothelial carcinoma (BLCA). However, a higher score showed significant associations with a better prognosis in BLCA patients. This would partly be attributed to the fact that a higher score is associated with more mutations occurring in these patients, as well as more treatment options. In addition, a potential regulation of phase separation by many mutations may lead to homeostasis related to better survival. Moreover, the mut_num variable was selected to predict the prognosis of breast cancer after stepwise regression modeling of the multivariable Cox regression model. Although the variable did not show statistical significance for prognosis when performing Kaplan‒Meier analysis using the median value as a cutoff, its combination with TMB, age, and sex indicated potential statistical significance. Overall, the survival analyses indicate the potential role of phase separation in patient prognosis.

In order to further understand the difference between mutations that positively regulate or negatively regulate protein phase separation, we then analyzed the pathogenicity of these phase separation-affecting mutations. We found that the positive mutations harbor a higher percent of mutations (35%) that are likely to be pathogenic than the negative mutations (29%). This would partially suggest the pathogenic function of promoting protein phase separation in cancer.

## 4. Discussion

Phase separation is reported to be relevant to cancer development [43]. Phase separation in cancer cells destroys the normal physiological function of phase-separating proteins and plays oncogenic roles in cancer [44]. Brief bioinformatics analysis showed that phase separation-related genes are mostly enriched in cancer rather than other diseases and also identified some pathways associated with cancer-related phase-separating proteins, including escaping programmed cell death and cell division control [45]. Our study also provided a similar conclusion that phase-separating proteins are mainly enriched in cell cycle-related pathways. Furthermore, one potential mechanism behind phase separation in cancer is aberrant chromatin looping [12]. This knowledge provides potential implications for phase separation in cancer therapy. However, the functional role of mutations in altering phase separation needs to be systematically evaluated in cancer [45].

We provided a systematic pan-cancer landscape to elucidate the role of phase separation in cancer development, which revealed the potential clues: (1) Mutations affecting the phase separation occupy a large part in all mutations, many are on PS regions, and some changed the PS regions of the corresponding wild-type proteins. (2) Genes harboring phase separation-affecting mutations are typically enriched in cell cycle-related pathways. (3) Features of phase separation-affecting mutations are associated with the prognosis of skin cutaneous melanoma, bladder cancer, and lung squamous cell carcinoma.

Previous studies have reported that phase separation commonly occurs in the synapse and is associated with neurological functions and processes [46]. In our study, we found that phase-separating protein-coding genes in cancers were commonly associated with brain development, including axon development, synapse organization, neuron-to-neuron synapses, etc. The mutual interaction of the nervous system and cancer has been given considerable attention [47]. In addition, we also found some other functions and pathways that potentially played roles in phase separation and cancer development. For example, we found that muscle-related functions were enriched in phase-separating protein-coding genes, while previous studies supported that cancer may cause muscle loss by overexpression of Pax7 [48], and the Pax7 protein was also predicted to be a phase-separating protein in this study. Moreover, two muscle-related proteins, FATZ and alpha-actinin-2, were found to be phase-separating proteins [49], while the alpha-actinin-2 protein was also revealed phase-separating protein in our analysis. These results may indicate the role of mutations in interfering with the phase separation of muscle development proteins, which is associated with muscle loss in cancer patients. Studies have indicated that phase separation is related to aging processes [50], and in the present study, we found an ACD protein whose function is to maintain telomere length and whose mutations cause aberrant phase transition. While telomere length is important for evaluating human aging, phase transition in the aging process may be one factor related to cancer development. However, further investigations about the associations of aging and phase separation are required to facilitate the understanding and treatment of cancer. Furthermore, previous studies indicated that the expression of phase-separating protein-coding genes has potential in skin cutaneous melanoma prognosis [18], bladder cancer molecular subtyping [19], and lung squamous cell carcinoma prognosis [51]. In this study, the comprehensive analysis of missense mutations affecting phase separation also indicated that the mutations are associated with the prognosis of the three types of cancer.

Previous studies also indicated mutations on disease-related genes, including AUTS2 (Autism Susceptibility Candidate 2), PRC1 (Polycomb Repressive Complex), and TP53 (Tumor Protein P53), are associated with aberrant phase separation. In this study, we identified phase separation regions on these proteins and found that mutations in these proteins exhibit aberrant changes in PS score. Despite its role in mediating post-translational modifications on histone tails to compact chromatin and inhibit gene expression [52], PRC1 can also activate gene expression by interacting with other predicted phase-separating proteins, including AUTS2 [53]. The mutations in AUTS2 IDRs may lead to altered condensate properties or phase separation threshold concentrations of PRC1-AUTS2 condensates [13]. In another way, the DNA-binding domain of mutant p53 (p53C) protein undergoes phase separation and forms aggregates with amyloid properties, which cause oncogenic gain-of-function (GoF) [54]. Furthermore, we defined aberrant mutations in the study and found that the genes carrying aberrant mutations are enriched in cell fate commitment, RNA splicing, and morphogenesis. Phase separation is reported to be related to RNA splicing [55] and cell fate commitment [56,57]. Therefore, our results suggest that the cancer mutations that changed the threshold concentration of phase separation are related to cell fate transition and RNA splicing.

In this study, we also found that mutations in non-phase separation regions could extend phase separation regions, which indicates potential regulation of phase separation for these mutations. For example, the p.N74S mutation on the ACD protein abruptly changes the PS score of the neighboring peptides of the ACD protein from 0.19 to 0.54, which results in an extension of PS regions. Moreover, we also found the R333C mutation on the tetramerization domain (TD) of the p53 protein, obtaining a PS score alteration of −0.058. This is consistent with a previous study [58], which suggested that an oncogenic mutation on the TD of the p53 protein could impair the formation of p53 condensate, thereby promoting cancer progression. The mutations on non-PS regions may be different from the mutations on PS regions in affecting protein phase separation, considering that PS regions are mainly IDR regions and play regulatory roles in phase separation, while non-PS regions (mainly structured domains) affect phase separation by changing the protein structure and thus mediating the protein–protein or protein–RNA interactions [59]. In accordance with this assumption, the enrichment analysis comparing genes of non-PS mutations with genes of PS mutations indicated different biological processes involved in the two kinds of mutations. Our findings provided evidence on the role of mutations affecting phase separation in cancer development.

There are limitations in this study. First, the predicted phase-separating proteins have not been validated by experiments; however, we demonstrated considerable accuracy in our previous study. Second, limited to current computational methods, our analysis is based on missense mutations, while other kinds of cancer mutations are also reported to be important in affecting phase separation. In summary, we presented a landscape of pan-cancer mutations affecting phase separation, which quantified the effects of over 1,200,000 mutations of 16 cancer types on phase separation. Our study will provide a rich data resource and provide future guidance on understanding phase separation and cancer mutations.

## 5. Conclusions

This study has provided a pan-cancer analysis on mutations and phase separation, identifying several clues on how mutations affect phase separation and cancer prognosis. The study has bridged the gap between mutations, phase separation and cancer prognosis, suggesting potential implications on cancer treatment and prognosis.

## Data Availability

All the data were downloaded from the TCGA database (https://xenabrowser.net/datapages/ (accessed on 1 December 2024).

## Supplementary Materials

The following supporting information can be downloaded at https://www.mdpi.com/article/10.3390/biology14101320/s1, Table S1: Basic information of analyzed sixteen cancer types; Figure S1: Violin Plot of PS score comparing benign mutations and cancer mutations; Figure S2: Pathway enrichment analysis of phase-separating protein-coding genes and non-phase-separation protein-coding genes in cancer dataset. (A) GO enrichment analysis of phase-separating protein-coding genes; (B) GO enrichment analysis of non-phase-separation protein-coding genes; (C) KEGG analysis of phase-separating protein-coding genes; (D) KEGG analysis of non-phase-separation protein-coding genes; Figure S3: Pathway enrichment analysis of genes harboring phase separation-affecting benign mutations. (A) GO analysis of genes harboring phase separation-affecting benign mutations; (B) KEGG analysis of genes harboring phase separation-affecting benign mutations; Figure S4: GO enrichment analysis of genes carrying most cancer mutations on non-PS regions and PS regions. (A) GO analysis of genes carrying most cancer mutations on non-PS regions; (B) GO analysis of genes carrying most cancer mutations on PS regions.
