# Supplementary material for: Pan-Cancer Analysis of Mutations Affecting Protein Liquid–Liquid Phase Separation Revealing Clinical Implications
Source: Biology (Basel). 2025 Sep 25;14(10):1320. doi: 10.3390/biology14101320 (PMC12562164; doi:10.3390/biology14101320)
Supplement: Supplementary file 1 [file biology-14-01320-s001.zip › biology-3853569-supplementary.pdf]

Supplementary Materials

Supplementary Table S1. Basic information of analyzed sixteen cancer type

Cancer Type	Abbreviation	Mutation Numbers	Protein Numbers
**Uterine Corpus Endometrial Carcinoma**	**UCEC**	403190	17498
**Skin Cutaneous Melanoma**	**SKCM**	201406	15867
**Colon adenocarcinoma**	**COAD**	127074	16139
**Lung adenocarcinoma**	**LUAD**	117121	15077
**Stomach adenocarcinoma**	**STAD**	106807	15490
**Lung squamous cell carcinoma**	**LUSC**	100672	14869
**Bladder Urothelial Carcinoma**	**BLCA**	73127	14588
**Breast invasive carcinoma**	**BRCA**	60392	13951
**Head and Neck squamous cell carcinoma**	**HNSC**	55258	13336
**Glioblastoma multiforme**	**GBM**	46728	12811
**Cervical squamous cell carcinoma and endocervical adenocarcinoma**	**CESC**	45669	13145
**Ovarian serous cystadenocarcinoma**	**OV**	38392	11762
**Rectum adenocarcinoma**	**READ**	34874	11359
**Liver hepatocellular carcinoma**	**LIHC**	27361	10469
**Esophageal carcinoma**	**ESCA**	19703	8625
**Brain Lower Grade Glioma**	**LGG**	19645	8802

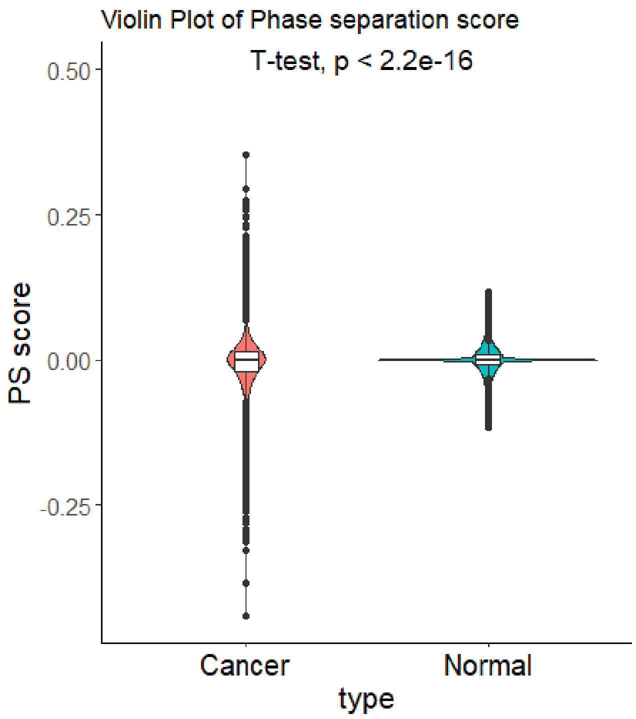

Supplementary Figure S1. Violin Plot of PS score comparing benign mutations and cancer mutations.

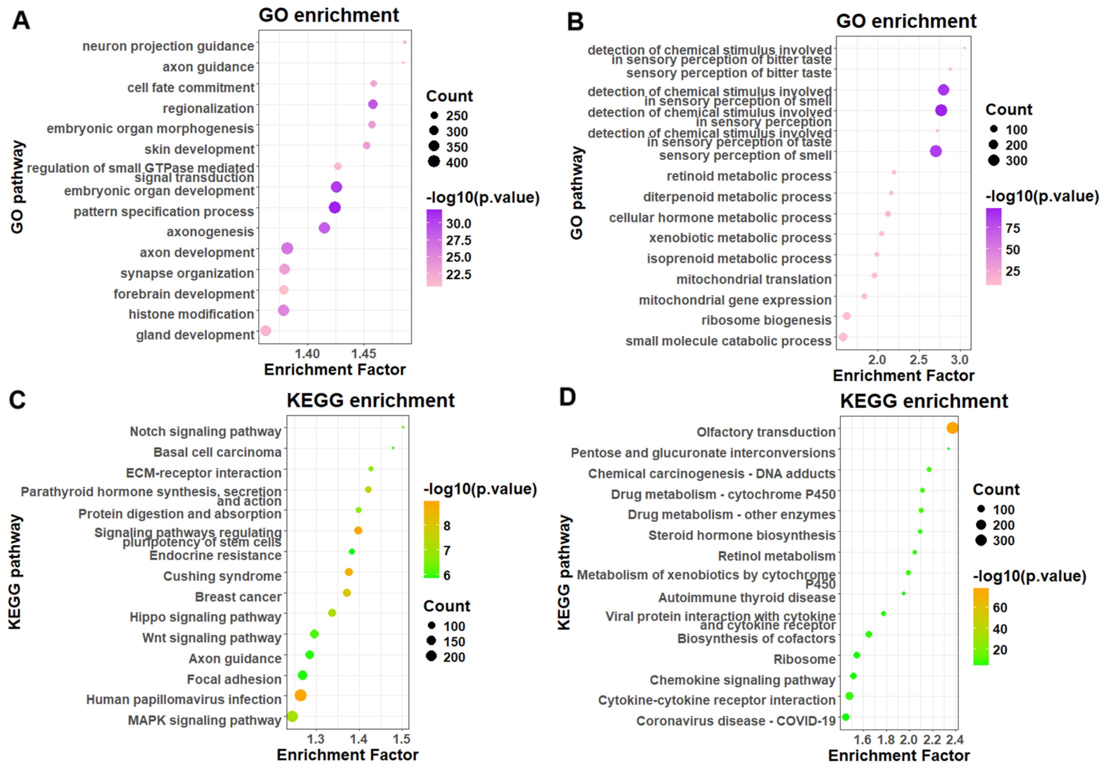

Supplementary Figure S2. Pathway enrichment analysis of phase separating protein-coding genes and non-phase-separation protein-coding genes in cancer dataset. (A) GO enrichment analysis of phase separating protein-coding genes; (B) GO enrichment analysis of non-phase-separation protein-coding genes; (C) KEGG analysis of phase separating protein-coding genes; (D) KEGG analysis of non-phase-separation protein-coding genes.

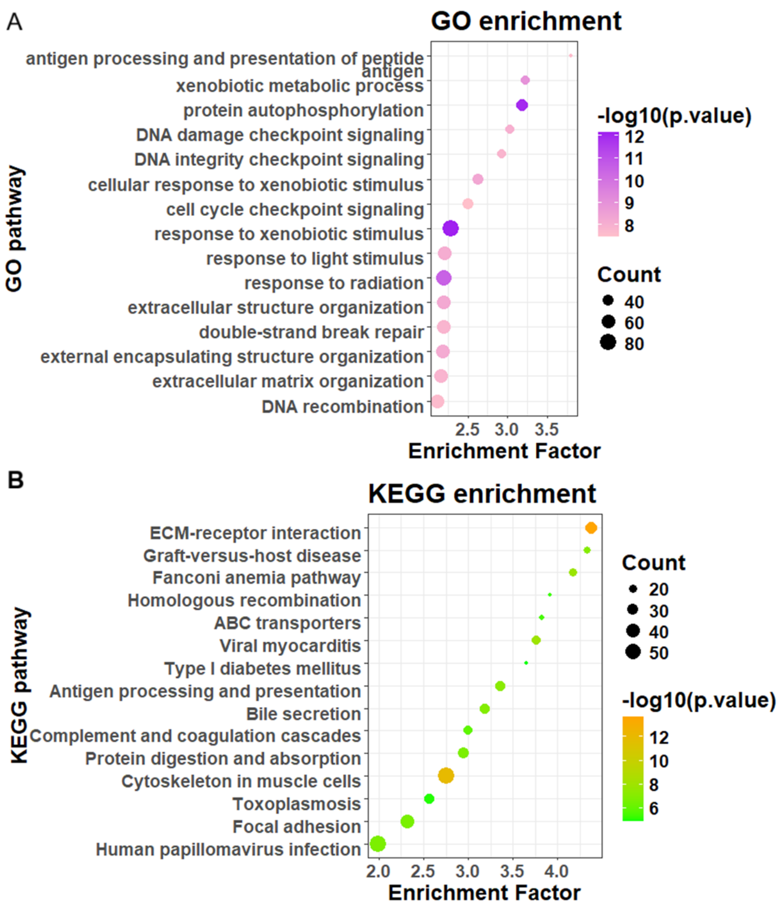

Supplementary Figure S3. Pathway enrichment analysis of genes haboring phase separation-affecting benign mutations. (A) GO analysis of genes harboring phase separation-affecting benign mutations; (B) KEGG analysis of genes harboring phase separation-affecting benign mutations.

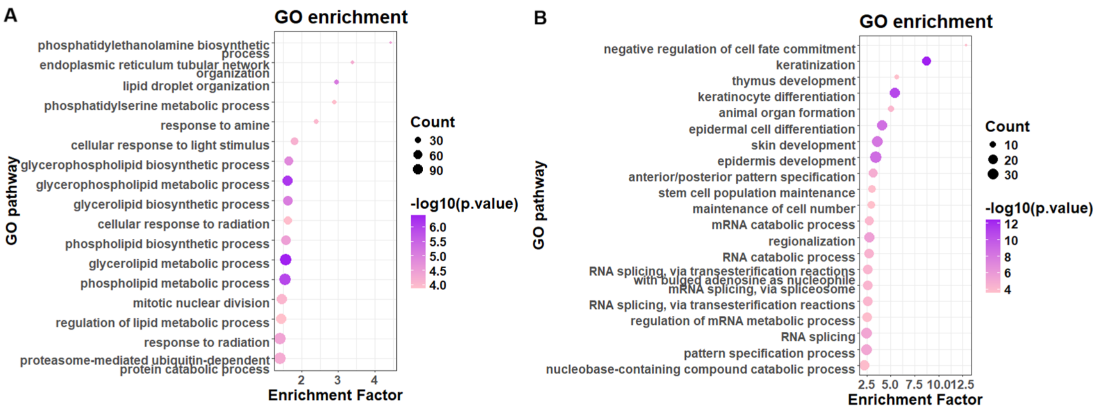

Supplementary Figure S4. GO enrichment analysis of genes carrying most cancer mutations on non-PS regions and PS regions. (A) GO analysis of genes carrying most cancer mutations on non-PS regions; (B) GO analysis of genes carrying most cancer mutations on PS regions.
